# Supplementary material for: Resistance to Diet Induced Visceral Fat Accumulation in C57BL/6NTac Mice Is Associated with an Enriched Lactococcus in the Gut Microbiota and the Phenotype of Immune B Cells in Intestine and Adipose Tissue
Source: Microorganisms. 2023 Aug 25;11(9):2153. doi: 10.3390/microorganisms11092153 (PMC10535569; doi:10.3390/microorganisms11092153)
Supplement: Supplementary file 1 [file microorganisms-11-02153-s001.zip › microorganisms-2509978-supplementary.pdf]

SUPPLENTARY FILES

SUPPLEMENTARY DATA

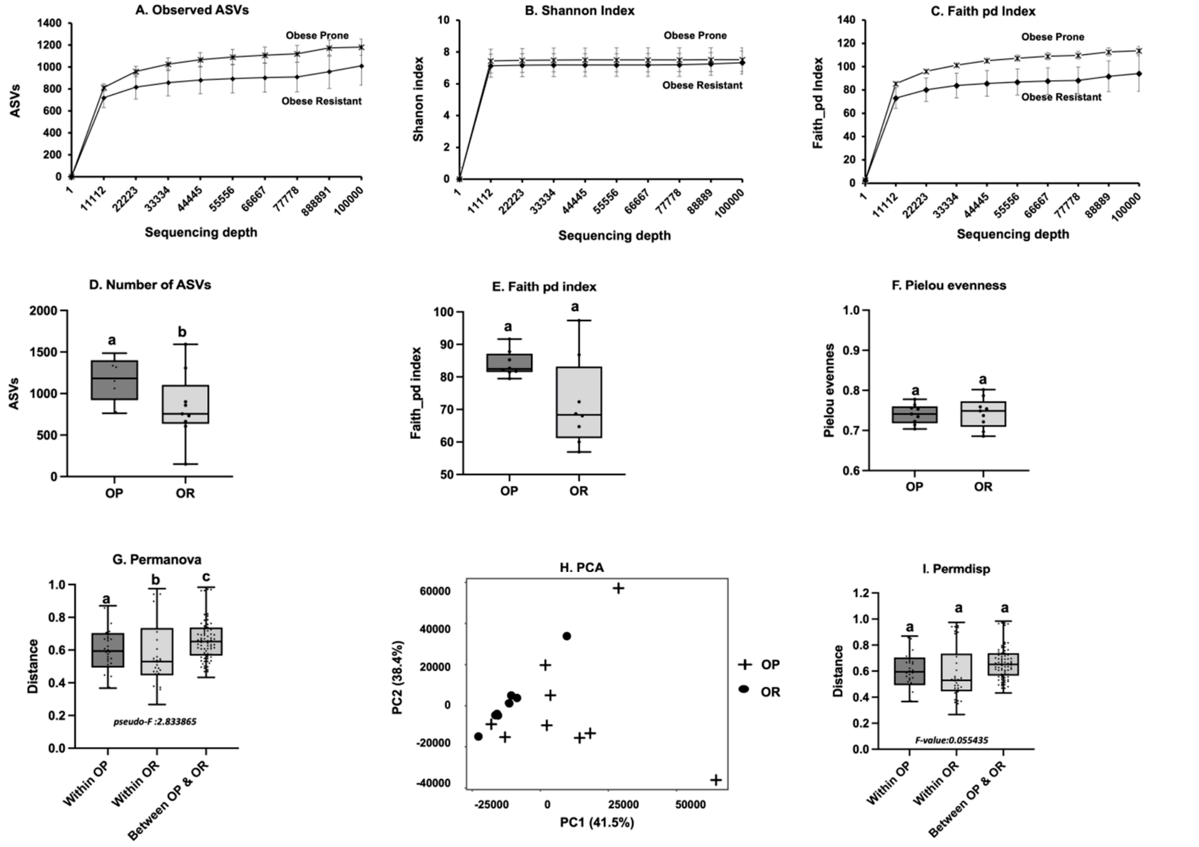

Supplementary Figure S1. Diversity measures among bacteria in the fecal contents of OR and OP mice

(A-C). Obesrved ASVs and Faith-pd were both higher in OP mice (P<0.01). The Shannon index did not show diffreneces (p=ns).

(D-F) Alpha diversity indices as detrmined by observed ASVs was higher in OP mice (P<0.05). The Faith-pd and the Pielou-e eveness indices were both not diffrenet between OP and OR (P=ns).

(G). The one-way Permanova non-parametric test of significant difference between groups, based on weighted unifrac distance showed significant differences between OP and OR bacteria composition (pseudo-F=2.83; P=0.007). The first and second boxplot is the intra group variability within OP or OR while the third box plot is the variability between the OP and OR groups.

(H). The visualization of the weighted unifrac distance between the OP and OR mice.

(I). Weighted unifrac Permdisp; the within group variation (dispersion) in the bacterial communities were not different between OP and OR mice (pseudo-F=0.056; P=ns).

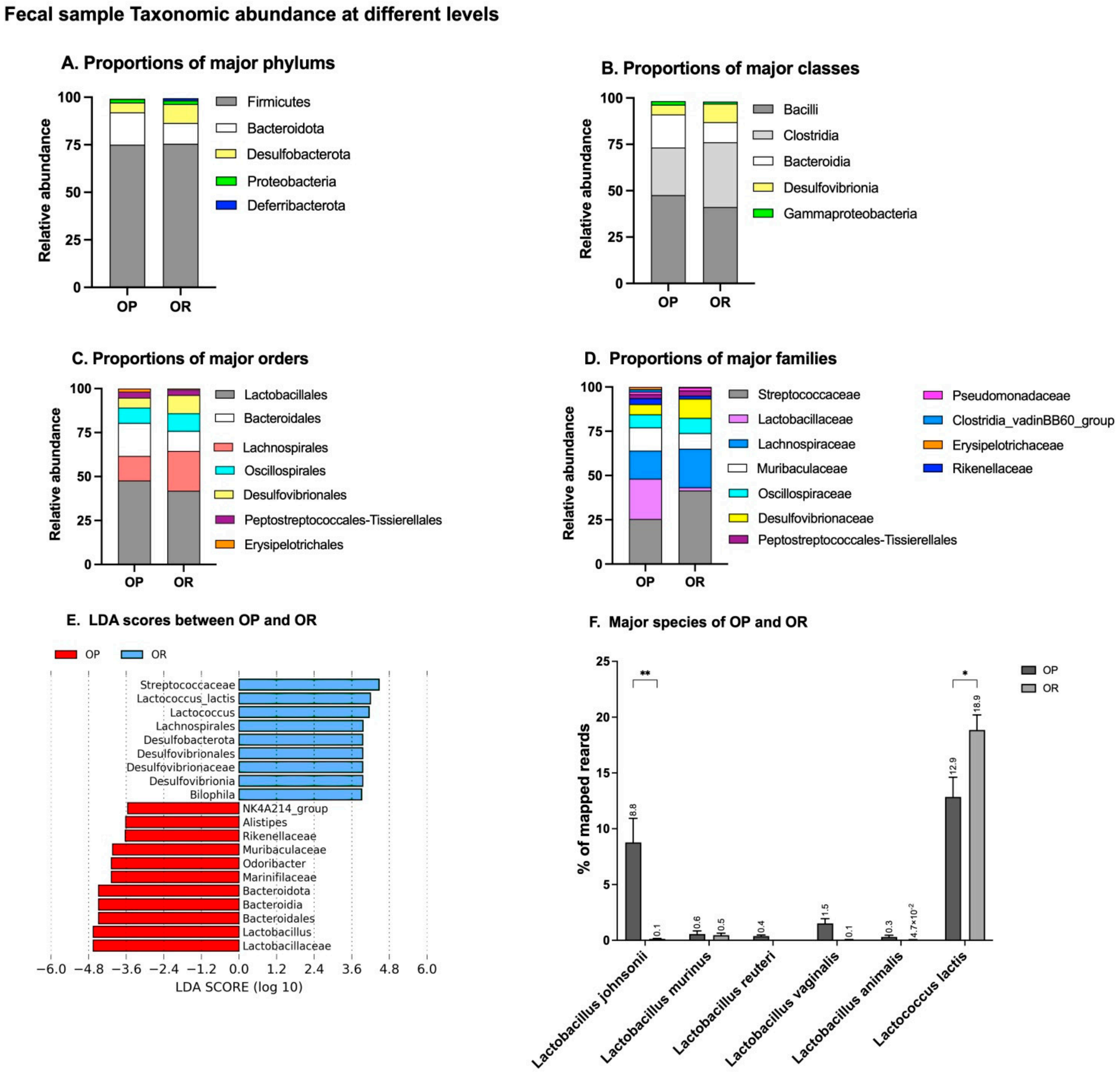

Supplementary Figure S2. The relative abundance of bacterial groups in fecal samples

(A). Abundance of Phyla Firmicutes and Proteobacteria was not different between OP and OR mice in the lower gut contents. Bacteroidota was higher in OR mice while Desulfobacteridota and Deferribacterota were significantly higher in OR mice.

(B). Classes Bacilli, Bacteroidia and Gammaproteobacteria were more abundant in OP mice while the classes Clostridia, Desulfobrionia, Deferribacteres were more abundant in OR mice.

(C). Orders Lactobacillales, Bacteroidales and Erysipelotrichales were more abundant higher in OP mice, while orders Lachnospirales, Oscillospirales and Desulfovibrionales were more abundant in OR mice.

(D). Families Lactobacillaceae, Erysipelotrichaceae, Muribaculaceae, Rikenellaceae were more abundant in OP mice while families Streptococcaceae, Desulvobrionaceae, Lachnospiraceae and Oscillospiraceae were more abundant in OR mice.

(E). Discriminative taxa selected based on an LDA score cutoff of 3.0 and ranked according to their effect size and statistically determined based on a Kruskal–Wallis and pairwise Wilcoxon tests at $P<0.05$. The

length of the histogram represents the LDA score; i.e., the degree of influence of species with significant difference between different groups.
(F). Proportions of Lactococcus and Lactobacillus species identified in OP and OR mice (*P<0.05; **P<0.01)

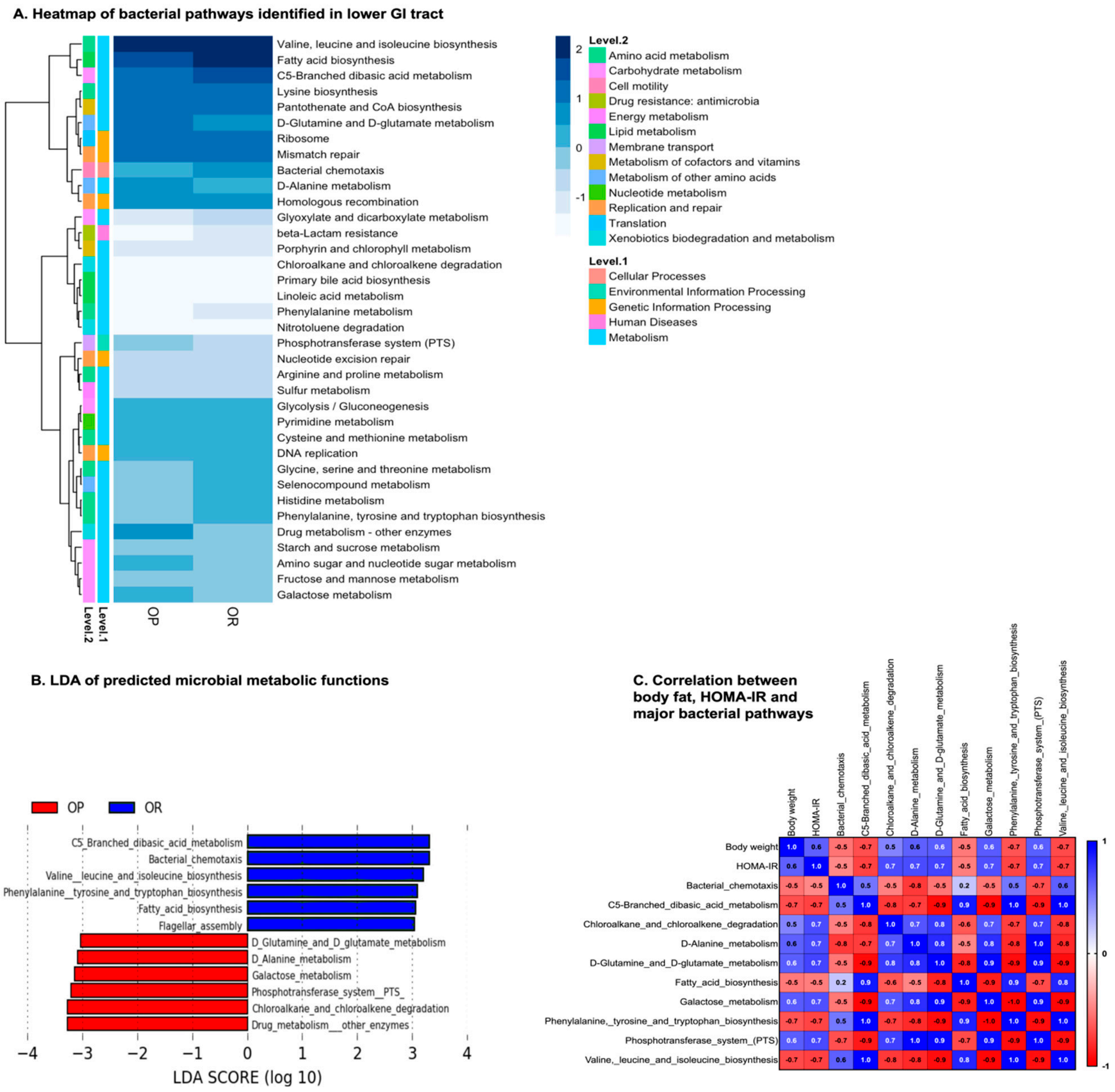

Supplementary Figure S3. Comparison of KEGG pathways predicted by PICRUSt in fecal samples

Predicted bacterial metabolic pathways from the Kyoto Encyclopedia of Genes and Genomes (KEGG) Ortholog (KO) database by PICRUSt2 using 16S rRNA gene sequences.
(A). Heatmap of 36 second-level classification KEGG pathways identified in the fecal contents.
(B). Histogram of the LDA scores calculated for the differentially abundant features in the fecal bacteria. Selection of discriminative microbial pathways between groups were based on an LDA score cutoff of 3.0 and differences in the relative abundances of pathway (converted to log base 10) were statistically determined based on a Kruskal–Wallis and pairwise Wilcoxon tests. A p-value of < 0.05 was considered

significant. The length of the histogram represents the LDA score; i.e., the degree of influence of species with significant difference between different groups

(C). Pearson correlation analysis of body fat and insulin resistance with major microbial pathways identified. Values displayed in the boxes are the correlation person coefficient 'r' values at $P<0.05$.

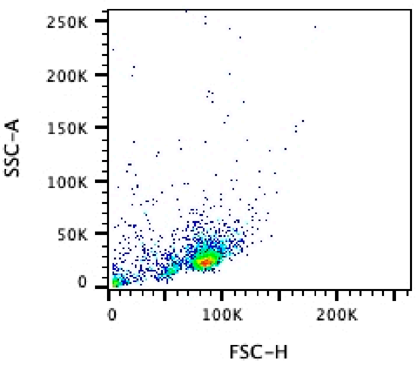

Unstained Control

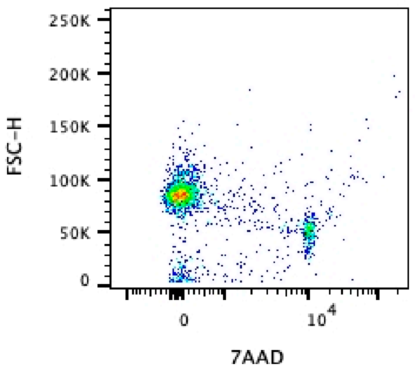

7AAD control for Live and Dead cells

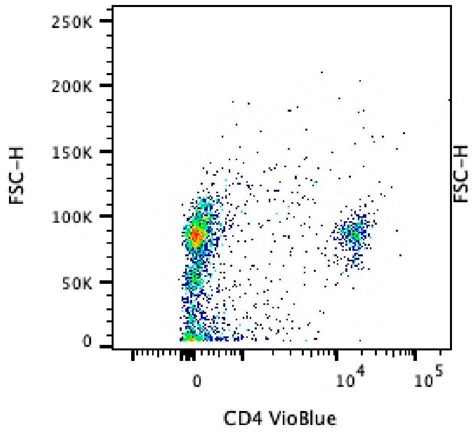

CD4 VioBlue control

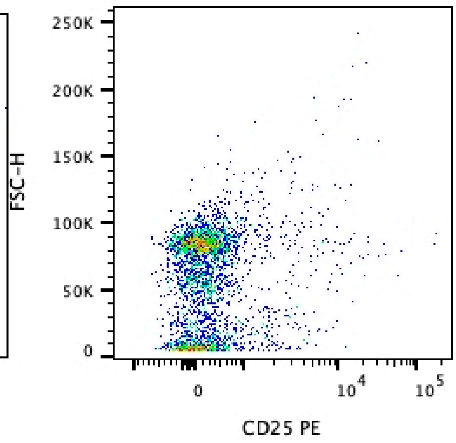

CD25 PE control

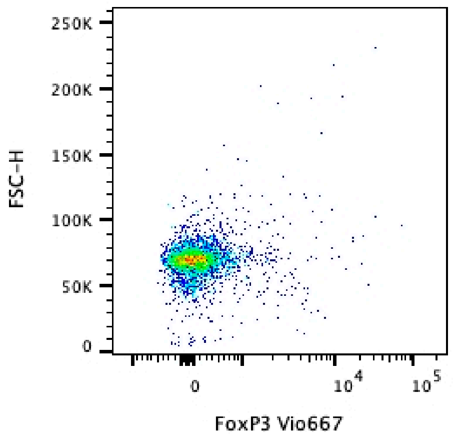

FoxP3 Vio667 control

Supplementary Figure S4A. Controls for Tregs

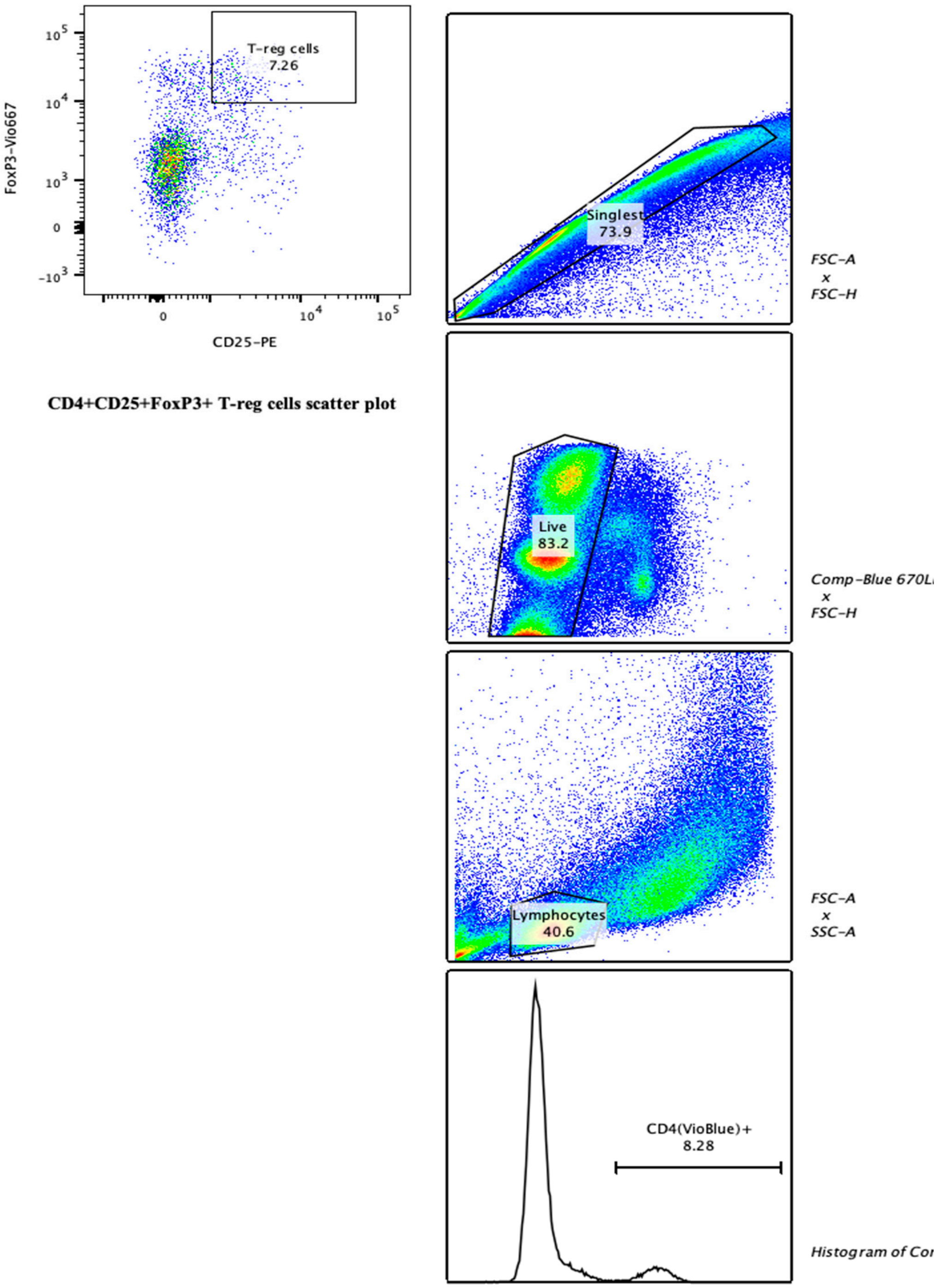

Supplementary Figure S4B. Scatter Plot for Tregs

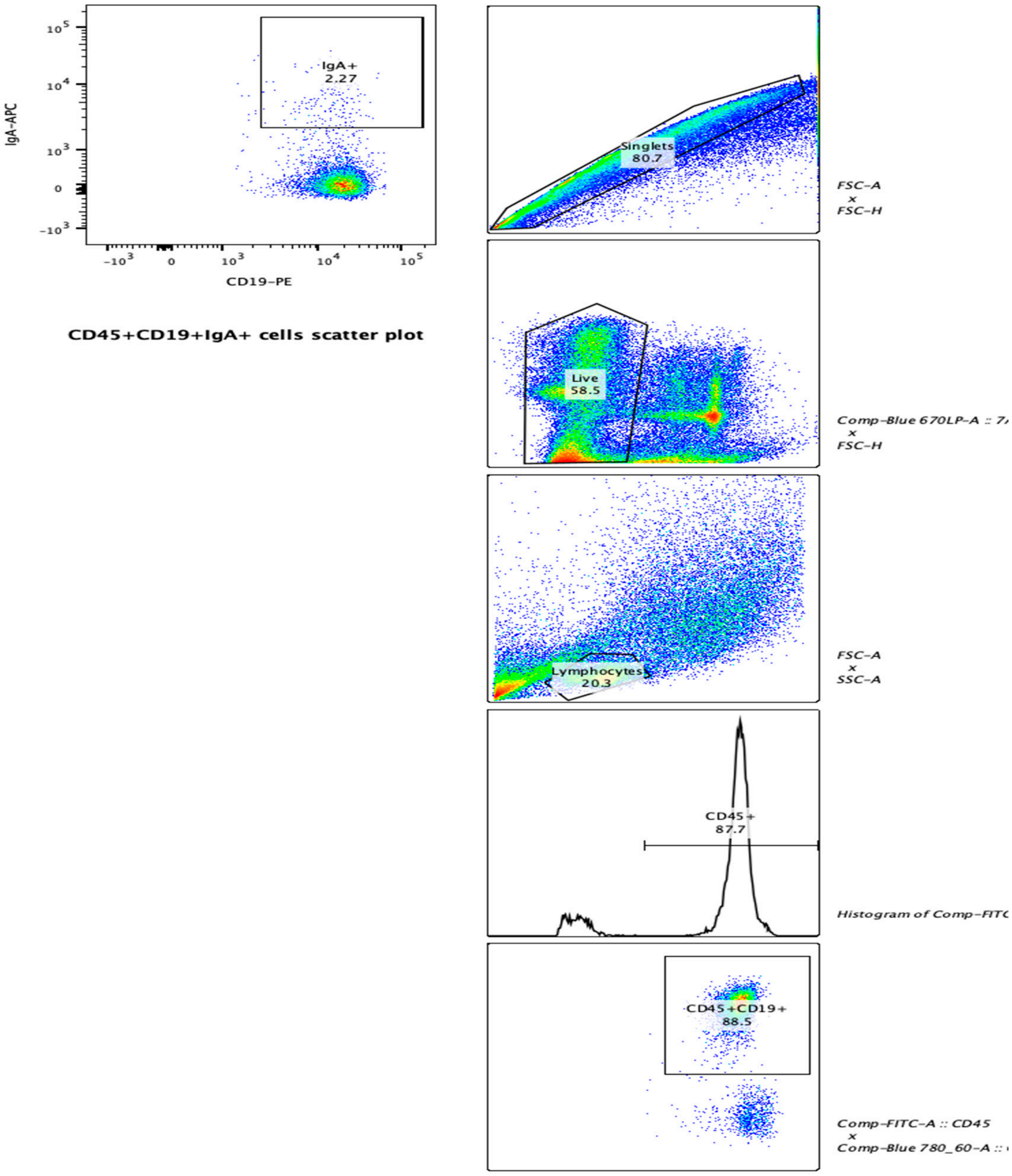

SUPPLEMENTARY Figure S4C. Controls for Tregs

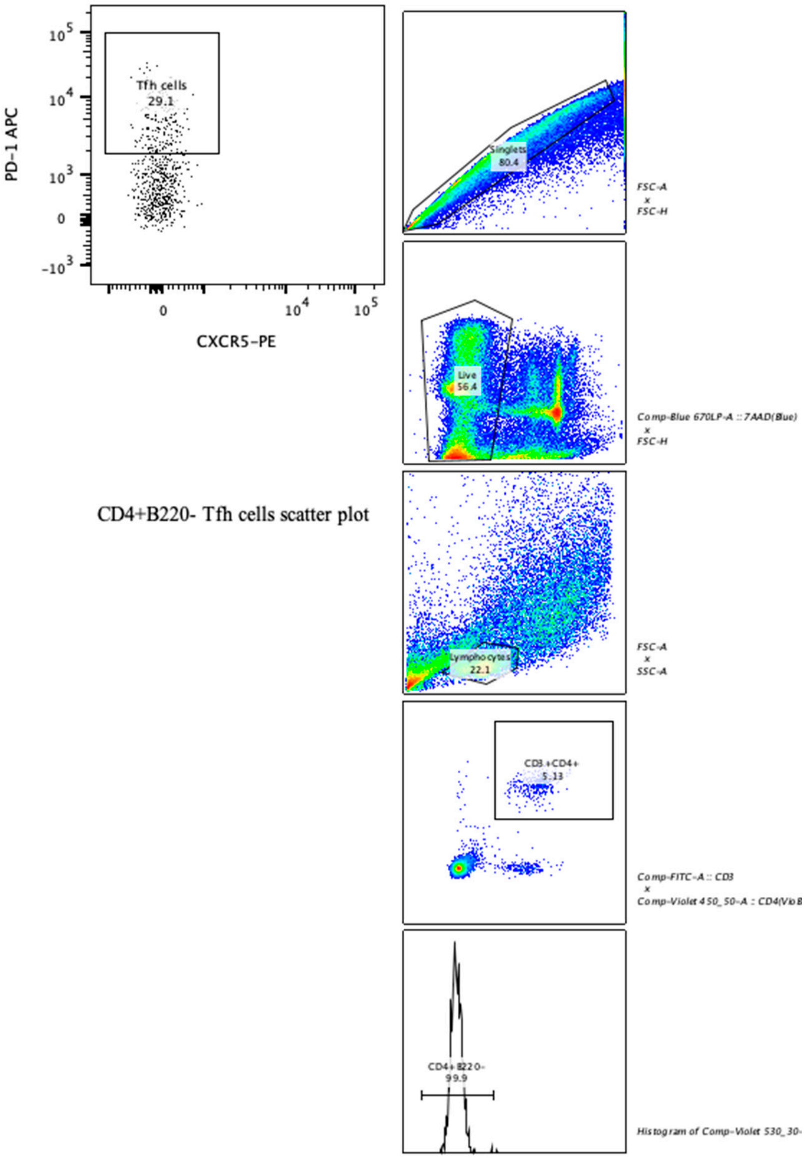

Supplementary Figure S5A. Scatter Plot for Intestinal B cells

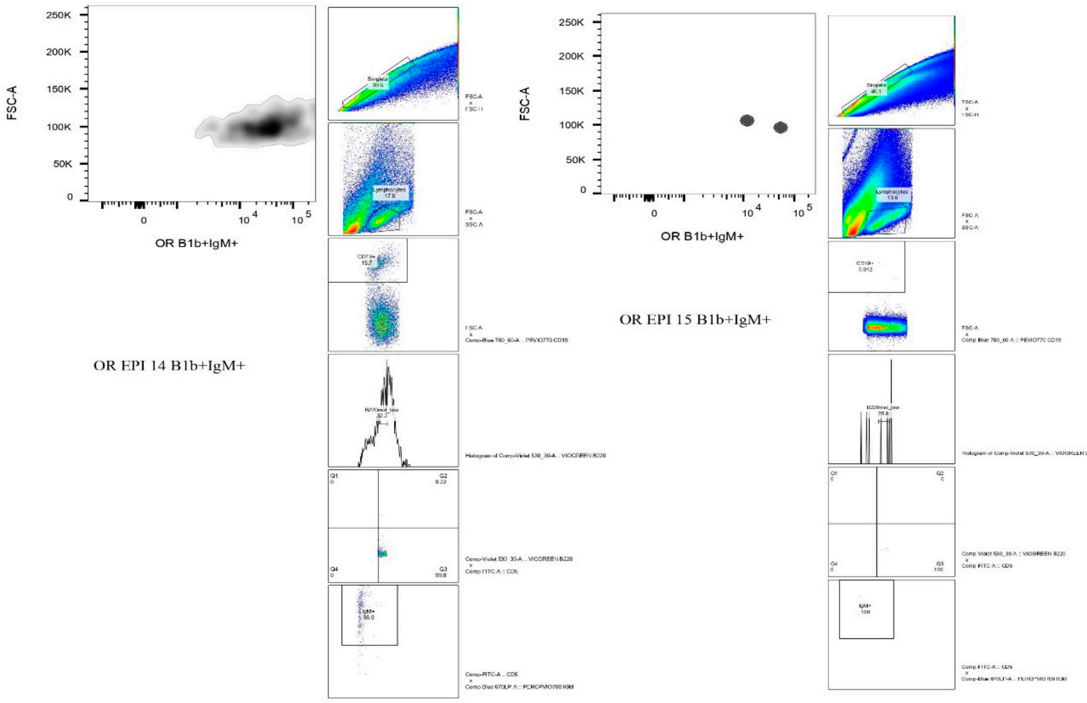

Supplementary Figure S5B. Scatter Plot for Adipose tissue B cells

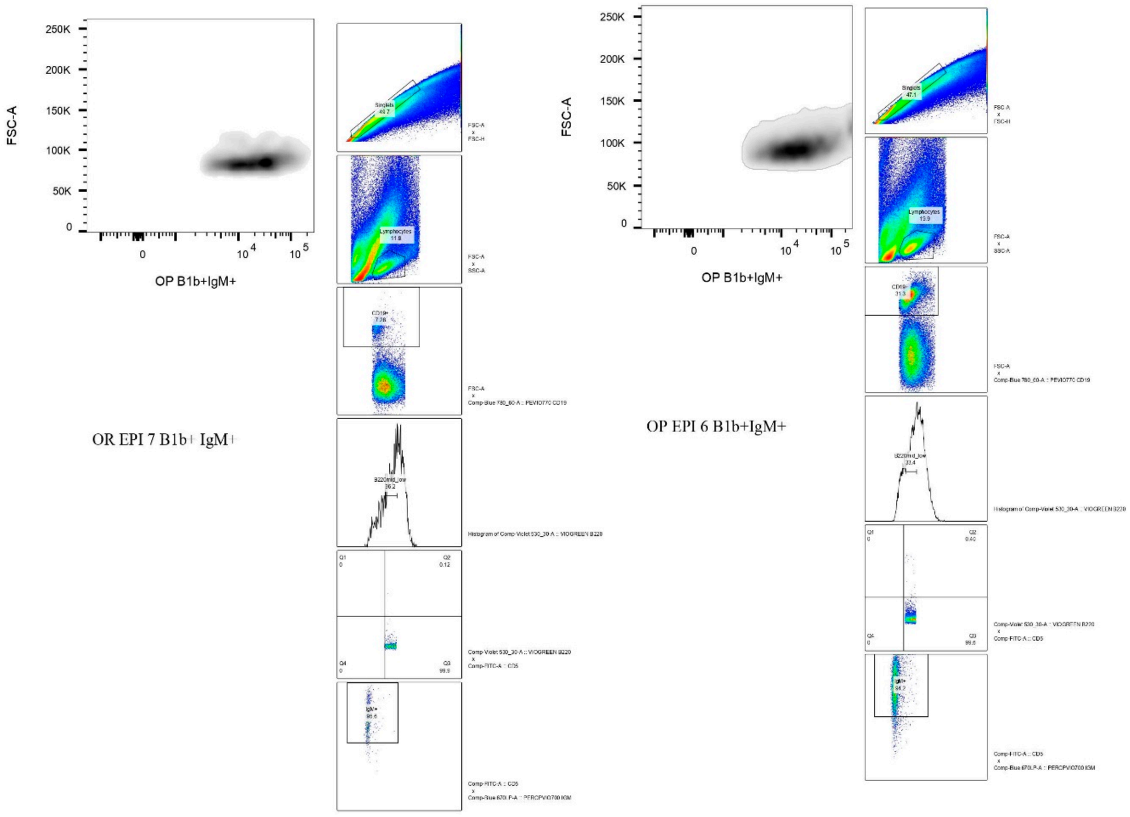

Supplementary Figure S5C. Scatter Plot for Adipose tissue B cells

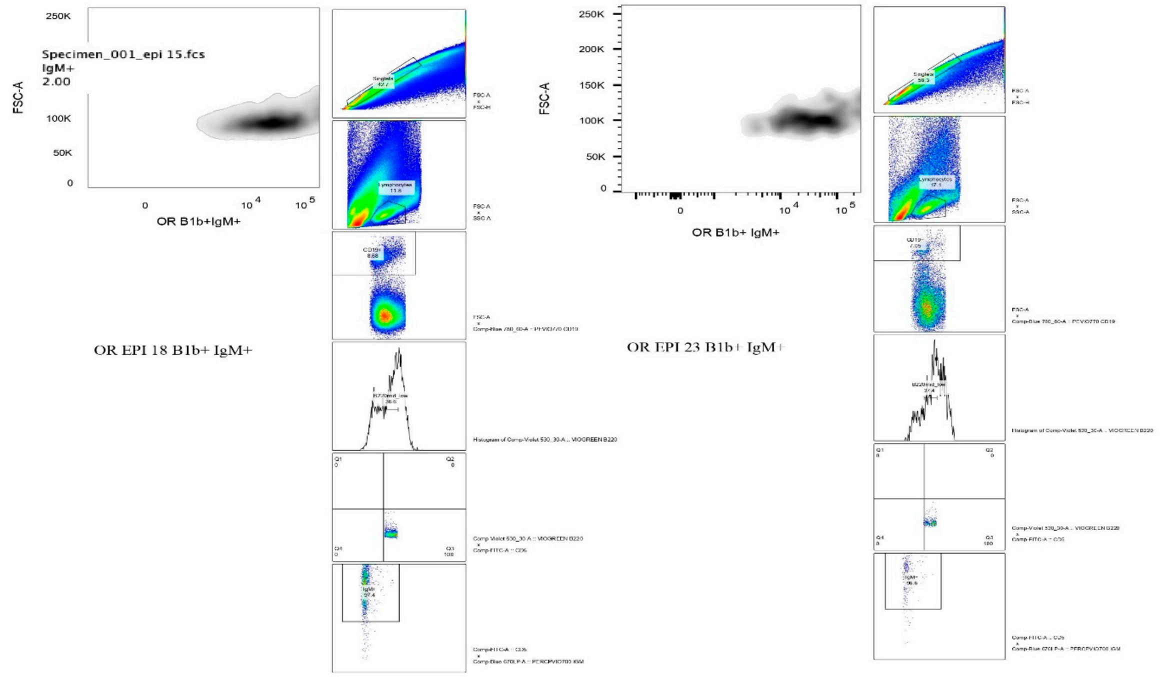

Supplementary Figure S5D. Scatter Plot for Adipose tissue B cells

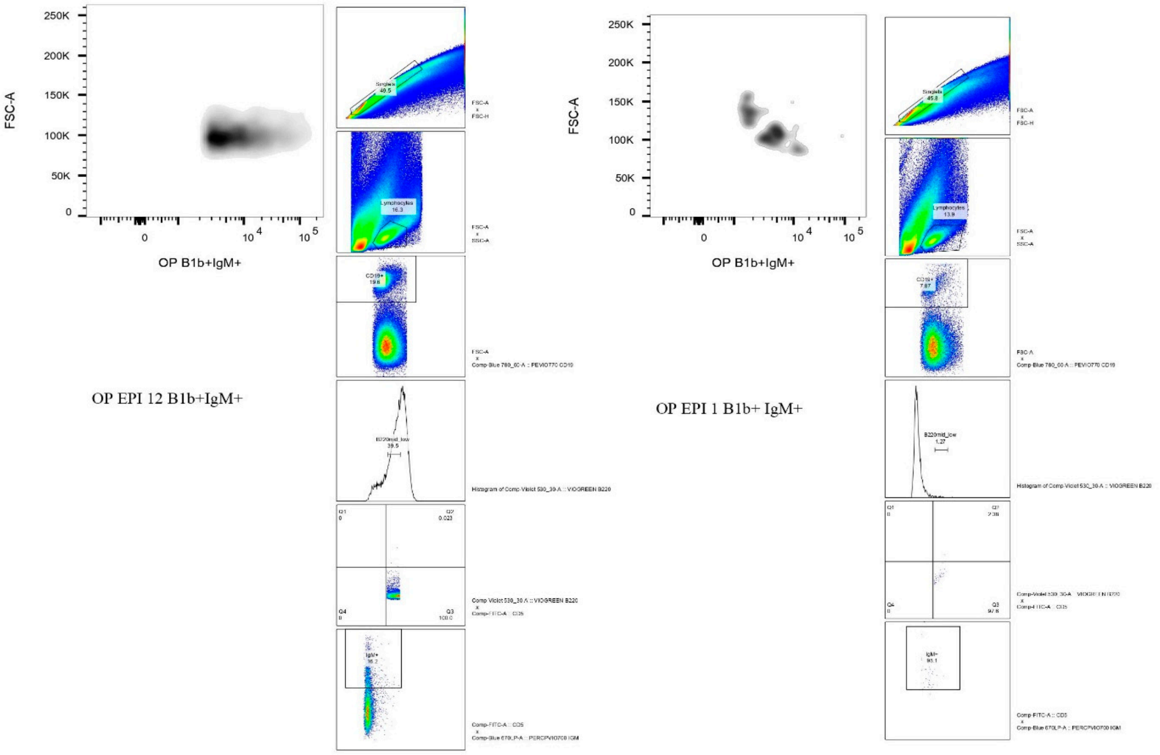

Supplementary Figure S5E. Scatter Plot for Adipose tissue B cells

SUPPLEMENTARY Table S1. Detailed Taxonomic Composition of intestinal contents

***Bold items indicate significant differences.**

Taxa	OP	OR	*P value*
**Actinobacteriota**			
P Actinobacteriota; C Coriobacteriia; O Coriobacteriales; F Eggerthellaceae	0.02	0.02	ns
P Actinobacteriota; C Coriobacteriia; O Coriobacteriales; F Eggerthellaceae; G Enterorhabdus; S uncultured_bacterium	0.06	0.07	ns
P Actinobacteriota; C Coriobacteriia; O Coriobacteriales; F Eggerthellaceae	0.08	0.08	ns
**Bacteroidota**			
**P Bacteroidota; C Bacteroidia; O Bacteroidales**	**0.73**	**3.62**	**0.03**
**P Bacteroidota; C Bacteroidia; O Bacteroidales; F Marinifilaceae; G Odoribacter**	**0.01**	**0.08**	**0.02**
**P Bacteroidota; C Bacteroidia; O Bacteroidales; F Muribaculaceae; G Muribaculaceae**	**0.64**	**2.88**	**0.04**
**P Bacteroidota; C Bacteroidia; O Bacteroidales; F Rikenellaceae; G Alistipes;**	**0.06**	**0.60**	**0.02**
P Bacteroidota; C Bacteroidia; O Bacteroidales; F Tannerellaceae; G Parabacteroides	0.01	0.04	ns
**Deferribacterota**			
P Deferribacterota; C Deferribacteres; O Deferribacterales; F Deferribacteraceae; G Mucispirillum; S uncultured_bacterium	0.00	0.09	ns
**Desulfobacterota**			
**P Desulfobacterota; C Desulfovibrionia; O Desulfovibrionales**	**0.79**	**3.73**	**0.05**
**P Desulfobacterota; C Desulfovibrionia; O Desulfovibrionales; F Desulfovibrionaceae; G Bilophila**	**0.28**	**3.02**	**0.03**
P Desulfobacterota; C Desulfovibrionia; O Desulfovibrionales; F Desulfovibrionaceae; G Desulfovibrio	0.29	0.28	ns
**Firmicutes**			

**P Firmicutes**	**98.10**	**91.04**	**0.04**
P Firmicutes; C Bacilli; O Lactobacillales	87.93	80.28	ns
**P Firmicutes; C Bacilli; O Lactobacillales; F Lactobacillaceae; G Lactobacillus**	**0.82**	**0.38**	**0.04**
**P Firmicutes; C Bacilli; O Lactobacillales; F Lactobacillaceae; G Lactobacillus; S uncultured_bacterium**	**36.81**	**7.82**	**0.01**
P Firmicutes; C Bacilli; O Lactobacillales; F Streptococcaceae	50.29	72.01	ns
P Firmicutes; C Bacilli; O Lactobacillales; F Streptococcaceae; G Lactococcus	10.59	13.43	ns
**P Firmicutes; C Bacilli; O Lactobacillales; F Streptococcaceae; G Lactococcus; S Lactococcus_lactis**	**39.50**	**58.25**	**0.04**
P Firmicutes; C Bacilli; O Lactobacillales; F Streptococcaceae; G Streptococcus	0.12	0.19	ns
P Firmicutes; C Bacilli; O Lactobacillales; F Streptococcaceae; G Streptococcus; S uncultured_bacterium	0.04	0.06	ns
P Firmicutes; C Bacilli; O Staphylococcales; F Staphylococcaceae; G Staphylococcus	0.01	0.02	ns
P Firmicutes; C Clostridia	7.58	10.40	ns
P Firmicutes; C Clostridia; O Clostridiales; F Clostridiaceae	0.03	1.03	ns
P Firmicutes; C Clostridia; O Clostridiales; F Clostridiaceae; G Candidatus_Arthromitus	0.02	0.55	ns
P Firmicutes; C Clostridia; O Clostridiales; F Clostridiaceae; G Candidatus_Arthromitus; S Candidatus_Arthromitus	0.00	0.04	ns
**P Firmicutes; C Clostridia; O Lachnospirales; F Lachnospiraceae**	**1.16**	**4.43**	**0.03**
P Firmicutes; C Clostridia; O Lachnospirales; F Lachnospiraceae; G [Ruminococcus]_torques_group	0.01	0.01	ns
P Firmicutes; C Clostridia; O Lachnospirales; F Lachnospiraceae; G Blautia	0.03	0.12	ns
**P Firmicutes; C Clostridia; O Lachnospirales; F Lachnospiraceae; G Blautia; S uncultured_bacterium**	**0.14**	**0.75**	**0.03**
P Firmicutes; C Clostridia; O Lachnospirales; F Lachnospiraceae; G Lachnoclostridium; S uncultured_bacterium	0.15	0.25	ns
P Firmicutes; C Clostridia; O Lachnospirales; F Lachnospiraceae; G Lachnospiraceae_NK4A136_group; S uncultured_bacterium	0.08	0.31	ns
P Firmicutes; C Clostridia; O Lachnospirales; F Lachnospiraceae; G Lachnospiraceae_UCG-006; S uncultured_bacterium	0.03	0.07	ns
**P Firmicutes; C Clostridia; O Lachnospirales; F Lachnospiraceae; G Roseburia; S uncultured_bacterium**	**0.08**	**0.56**	**0.01**
P Firmicutes; C Clostridia; O Lachnospirales; F Lachnospiraceae; G Tuzzerella; S uncultured_bacterium	0.02	0.05	ns
**P Firmicutes; C Clostridia; O Lachnospirales; F Lachnospiraceae; G uncultured; S uncultured_bacterium**	**0.32**	**1.51**	**0.02**
P Firmicutes; C Clostridia; O Oscillospirales	0.33	1.99	ns
P Firmicutes; C Clostridia; O Oscillospirales; F Oscillospiraceae	0.04	0.38	ns

P Firmicutes; C Clostridia; O Oscillospirales; F Oscillospiraceae; G Colidextribacter	0.04	0.12	ns
**P Firmicutes; C Clostridia; O Oscillospirales; F Oscillospiraceae; G Colidextribacter; S uncultured_bacterium**	**0.07**	**0.59**	**0.05**
**P Firmicutes; C Clostridia; O Oscillospirales; F Oscillospiraceae; G uncultured**	**0.01**	**0.15**	**0.02**
P Firmicutes; C Clostridia; O Oscillospirales; F Oscillospiraceae; G uncultured; S uncultured_bacterium	0.15	0.55	ns
P Firmicutes; C Clostridia; O Oscillospirales; F Ruminococcaceae; G Anaerotruncus	0.00	0.01	ns
P Firmicutes; C Clostridia; O Oscillospirales; F Ruminococcaceae; G Anaerotruncus; S uncultured_bacterium	0.01	0.05	ns
P Firmicutes; C Clostridia; O Oscillospirales; F Ruminococcaceae; G Incertae_Sedis; S uncultured_bacterium	0.01	0.04	ns
P Firmicutes; C Clostridia; O Oscillospirales; F Ruminococcaceae; G uncultured; S uncultured_bacterium	0.02	0.09	ns
P Firmicutes; C Clostridia; O Peptostreptococcales-Tissierellales; F Peptostreptococcaceae	5.70	2.61	ns
**P Firmicutes; C Clostridia; O Peptostreptococcales-Tissierellales; F Peptostreptococcaceae; G Paraclostridium**	**3.48**	**0.24**	**0.01**
**P Firmicutes; C Clostridia; O Peptostreptococcales-Tissierellales; F Peptostreptococcaceae; G Romboutsia;**	**1.90**	**0.03**	**0.01**
**P Firmicutes; C Clostridia; O Peptostreptococcales-Tissierellales; F Peptostreptococcales-Tissierellales**	**0.32**	**2.33**	**0.00**

**Proteobacteria**			
P Proteobacteria; C Gammaproteobacteria	0.03	0.57	ns
**P Proteobacteria; C Gammaproteobacteria; O Enterobacterales; F Enterobacteriaceae**	**0.01**	**0.04**	**0.05**
P Proteobacteria; C Gammaproteobacteria; O Enterobacterales; F Enterobacteriaceae; G Escherichia-Shigella; S Escherichia_coli	0.01	0.02	ns
P Proteobacteria; C Gammaproteobacteria; O Enterobacterales; F Morganellaceae; G Proteus	0.01	0.02	ns
P Proteobacteria; C Gammaproteobacteria; O Enterobacterales; F Morganellaceae; G Proteus; S Proteus_mirabilis	0.01	0.04	ns
P Proteobacteria; C Gammaproteobacteria; O Orbales; F Orbaceae; G Gilliamella; S uncultured_gamma	0.00	0.45	ns

SUPPLEMENTARY Table S2. Detailed Taxonomic Composition of Fecal contents
***Bold items indicate significant differences.**

Taxa	OP	OR	*P value*
**Actinobacteriota**			
P Actinobacteriota; C Coriobacteriia; O Coriobacteriales	0.31	0.15	ns
**P Actinobacteriota; C Coriobacteriia; O Coriobacteriales;F Eggerthellaceae**	**0.06**	**0.01**	**0.042**
**P Actinobacteriota; C Coriobacteriia; O Coriobacteriales;F Eggerthellaceae;G Enterorhabdus**	**0.23**	**0.10**	**0.044**
**Bacteroidota**			
**P Bacteroidota; C Bacteroidia; O Bacteroidales**	**33.40**	**21.50**	**0.001**
P Bacteroidota; C Bacteroidia; O Bacteroidales;F Bacteroidaceae;G Bacteroides	1.50	0.29	ns
**P Bacteroidota; C Bacteroidia; O Bacteroidales;F Marinifilaceae;G Odoribacter;**	**12.60**	**8.72**	**0.005**
**P Bacteroidota; C Bacteroidia; O Bacteroidales;F Muribaculaceae;G Muribaculaceae;**	**11.70**	**8.18**	**0.004**
**P Bacteroidota; C Bacteroidia; O Bacteroidales;F Rikenellaceae;G Alistipes**	**3.07**	**1.64**	**0.010**
**P Bacteroidota; C Bacteroidia; O Bacteroidales;F Rikenellaceae;G Alistipes;S Alistipes_finegoldii**	**0.14**	**0.07**	**0.023**
P Bacteroidota; C Bacteroidia; O Bacteroidales;F Tannerellaceae;G Parabacteroides	0.55	0.45	ns
**Deferribacterota**			
**P Deferribacterota; C Deferribacteres; O Deferribacterales;F Deferribacteraceae;G Mucispirillum;**	**0.20**	**1.16**	**0.028**
**Desulfobacterota**			
**P Desulfobacterota; C Desulfovibrionia; O Desulfovibrionales;F Desulfovibrionaceae**	**5.16**	**9.97**	**0.015**
**P Desulfobacterota; C Desulfovibrionia; O Desulfovibrionales;F Desulfovibrionaceae;G Bilophila**	**3.02**	**7.30**	**0.006**
P Desulfobacterota; C Desulfovibrionia; O Desulfovibrionales;F Desulfovibrionaceae;G Desulfovibrio	1.12	1.48	ns
**Firmicutes**			
P Firmicutes	74.30	75.40	ns
P Firmicutes; C Bacilli	47.60	41.20	ns

P Firmicutes; C Bacilli; O Erysipelotrichales	1.57	0.67	ns
P Firmicutes; C Bacilli; O Erysipelotrichales; F Erysipelatoclostridiaceae;G Erysipelatoclostridium	0.10	0.16	ns
P Firmicutes; C Bacilli; O Lactobacillales	43.30	43.40	ns
P Firmicutes; C Bacilli; O Lactobacillales;F Lactobacillaceae;G Lactobacillus	0.90	0.27	ns
**P Firmicutes; C Bacilli; O Lactobacillales;F Lactobacillaceae;G Lactobacillus;S**	**19.50**	**1.47**	**0.002**
**P Firmicutes; C Bacilli; O Lactobacillales;F Streptococcaceae**	**22.80**	**38.40**	**0.002**
**P Firmicutes; C Bacilli; O Lactobacillales;F Streptococcaceae;G Lactococcus**	**6.74**	**14.10**	**0.002**
**P Firmicutes; C Bacilli; O Lactobacillales;F Streptococcaceae;G Lactococcus;S Lactococcus_lactis**	**15.90**	**24.10**	**0.004**
P Firmicutes; C Bacilli; O Lactobacillales;F Streptococcaceae;G Streptococcus	0.01	0.03	ns
**P Firmicutes; C Bacilli; O RF39;F RF39;G RF39;**	**0.47**	**0.03**	**0.023**
**P Firmicutes; C Clostridia**	**25.60**	**34.90**	**0.026**
P Firmicutes; C Clostridia; O Christensenellales;F Christensenellaceae;G Christensenellaceae_R-7_group	0.03	0.05	ns
P Firmicutes; C Clostridia; O Clostridia_UCG-014;F Clostridia_UCG-014;G Clostridia_UCG-014	0.71	0.28	ns
**P Firmicutes; C Clostridia; O Clostridia_vadinBB60_group;F Clostridia_vadinBB60_group;G Clostridia_vadinBB60_group**	**1.20**	**0.28**	**0.013**
P Firmicutes; C Clostridia; O Clostridiales;F Clostridiaceae;G Candidatus_Arthromitus	0.01	0.05	ns
**P Firmicutes; C Clostridia; O Lachnospirales;F Lachnospiraceae**	**14.20**	**20.20**	**0.048**
P Firmicutes; C Clostridia; O Lachnospirales;F Lachnospiraceae;G [Eubacterium]_xylanophilum_group;	0.02	0.00	ns
**P Firmicutes; C Clostridia; O Lachnospirales;F Lachnospiraceae;G [Ruminococcus]_torques_group**	**0.07**	**0.13**	**0.049**
P Firmicutes; C Clostridia; O Lachnospirales;F Lachnospiraceae;G ASF356	0.02	0.00	ns
P Firmicutes; C Clostridia; O Lachnospirales;F Lachnospiraceae;G Blautia	0.48	0.73	ns
P Firmicutes; C Clostridia; O Lachnospirales;F Lachnospiraceae;G Blautia	1.68	2.65	ns
**P Firmicutes; C Clostridia; O Lachnospirales;F Lachnospiraceae;G GCA-900066575;**	**0.09**	**0.18**	**0.048**
**P Firmicutes; C Clostridia; O Lachnospirales;F Lachnospiraceae;G Lachnoclostridium**	**0.01**	**0.07**	**0.016**
P Firmicutes; C Clostridia; O Lachnospirales;F Lachnospiraceae;G Lachnospiraceae_FCS020_group;	0.03	0.08	ns
P Firmicutes; C Clostridia; O Lachnospirales;F Lachnospiraceae;G Lachnospiraceae_NK4A136_group	0.02	0.01	ns
P Firmicutes; C Clostridia; O Lachnospirales;F Lachnospiraceae;G Lachnospiraceae_NK4A136_group;	1.46	1.65	ns
P Firmicutes; C Clostridia; O Lachnospirales;F Lachnospiraceae;G Lachnospiraceae_UCG-006;	0.20	0.36	ns
P Firmicutes; C Clostridia; O Lachnospirales;F Lachnospiraceae;G Lachnospiraceae_UCG-008;	0.02	0.00	ns
P Firmicutes; C Clostridia; O Lachnospirales;F Lachnospiraceae;G Roseburia	0.01	0.00	ns
P Firmicutes; C Clostridia; O Lachnospirales;F Lachnospiraceae;G Roseburia;	1.54	1.93	ns
**P Firmicutes; C Clostridia; O Lachnospirales;F Lachnospiraceae;G Tuzzerella;**	**0.36**	**0.86**	**0.009**
P Firmicutes; C Clostridia; O Lachnospirales;F Lachnospiraceae;G uncultured	0.04	0.10	ns
P Firmicutes; C Clostridia; O Lachnospirales;F Lachnospiraceae;G uncultured;	3.76	5.19	ns
P Firmicutes; C Clostridia; O Oscillospirales	7.95	9.61	ns
P Firmicutes; C Clostridia; O Oscillospirales;F Oscillospiraceae	3.75	4.04	ns

P Firmicutes; C Clostridia; O Oscillospirales;F Oscillospiraceae;G Colidextribacter	0.65	0.85	ns
P Firmicutes; C Clostridia; O Oscillospirales;F Oscillospiraceae;G Colidextribacter;	1.79	1.99	ns
P Firmicutes; C Clostridia; O Oscillospirales;F Oscillospiraceae;G NK4A214_group;	0.02	0.01	ns
P Firmicutes; C Clostridia; O Oscillospirales;F Oscillospiraceae;G Oscillibacter;	0.09	0.08	ns
P Firmicutes; C Clostridia; O Oscillospirales;F Ruminococcaceae	1.68	2.12	ns
P Firmicutes; C Clostridia; O Oscillospirales;F Ruminococcaceae;G Anaerotruncus	0.16	0.27	ns
P Firmicutes; C Clostridia; O Oscillospirales;F Ruminococcaceae;G Incertae_Sedis;	0.11	0.08	ns
P Firmicutes; C Clostridia; O Oscillospirales;F Ruminococcaceae;G Negativibacillus;	0.03	0.01	ns
**P Firmicutes; C Clostridia; O Oscillospirales;F UCG-010;G UCG-010;**	**0.11**	**0.02**	**0.005**
P Firmicutes; C Clostridia; O Peptococcales;F Peptococcaceae;	0.29	0.55	ns
P Firmicutes; C Clostridia; O Peptostreptococcales-Tissierellales	3.07	2.79	ns
P Firmicutes; C Clostridia; O Peptostreptococcales-Tissierellales;F Anaerovoracaceae;G [Eubacterium]_nodatum_group;	0.06	0.00	ns
**P Firmicutes; C Clostridia; O Peptostreptococcales-Tissierellales;F Peptostreptococcaceae;G Paraclostridium**	**0.19**	**0.00**	**0.031**
**P Firmicutes; C Clostridia; O Peptostreptococcales-Tissierellales;F Peptostreptococcaceae;G Romboutsia**	**0.74**	**0.00**	**0.033**
P Firmicutes; C Clostridia; O Peptostreptococcales-Tissierellales;F Peptostreptococcales-Tissierellales	2.02	2.72	ns
**Proteobacteria**			
P Proteobacteria; C Alphaproteobacteria	**0.11**	**0.02**	ns
**P Proteobacteria; C Alphaproteobacteria; O Rhodospirillales;F uncultured;G uncultured**	**0.08**	**0.01**	**0.026**
P Proteobacteria; C Gammaproteobacteria	1.90	1.12	ns
P Proteobacteria; C Gammaproteobacteria; O Burkholderiales	0.08	0.01	ns
**P Proteobacteria; C Gammaproteobacteria; O Enterobacterales**	**0.09**	**0.32**	**0.003**
**P Proteobacteria; C Gammaproteobacteria; O Enterobacterales;F Enterobacteriaceae**	**0.01**	**0.17**	**0.016**
P Proteobacteria; C Gammaproteobacteria; O Enterobacterales;F Enterobacteriaceae;G Escherichia-Shigella	0.03	0.01	ns
P Proteobacteria; C Gammaproteobacteria; O Enterobacterales;F Enterobacteriaceae;G Escherichia-Shigella;S Escherichia_coli	0.06	0.13	ns
P Proteobacteria; C Gammaproteobacteria; O Orbales;F Orbaceae;G Gilliamella;S uncultured_gamma	0.24	0.00	ns
P Proteobacteria; C Gammaproteobacteria; O Pseudomonadales;F Pseudomonadaceae;G Pseudomonas	1.28	1.53	ns

*Bold items indicate significant differences.

Supplementary Table S3. Primers used for qPCR analysis

Gene	Forward	Reverse
TLR-4	AGT GCC CCG CTT TCA CCT CT	TCC GGC TCT TGT GGA AGC CT
CD36	CAG TTG CTC CAC ACA TTT CAG	TCT CCT TCA ACA GTC TCC CT
MUC 2	TCA AAG TGC TCT CCA AAC TCT C	CCT CTC AGA ATT CCA CAC TCT T
Claudin-1	GTT TGC AGA GAC CCC ATC AC	AGA AGC CAG GAT GAA ACC CA
Occludin	CTC CCA TCC GAG TTT CAG GT	GCT GTC GCC TAA GGA AAG AG
F4/80	GGA AGG AAA TGG AGA GAA AG	GAA GAT CTA CCC TGG TGA AT
B-Actin	CCA GAG CAA GAG AGG TAT CC	CTG TGG TGG TGA AGC TGT AG
